# Procalcitonin-Guided Antimicrobial Therapy: Current Evidence for Clinical Decision-Making and Practical Limitations

**DOI:** 10.7759/cureus.116457

**Published:** 2026-09-17

**Authors:** Sumayyah Alshammari, Basmah Alrawaili, Roaa Alshammari, Saja Alruwaili, Reem Alruwaili, Abdulaziz Alzahrani, Alhassan Alzahrani

**Affiliations:** 1 College of Medicine, Northern Border University, Arar, SAU; 2 College of Medicine, King Abdulaziz University, Jeddah, SAU; 3 Department of Family Medicine, King Fahad Hospital Jeddah, Jeddah, SAU

**Keywords:** antibiotic decision-making, antibiotic stewardship, bacterial infections, biomarkers, procalcitonin

## Abstract

Antimicrobial resistance and unnecessary antibiotic exposure remain significant problems in clinical practice. Procalcitonin (PCT) has emerged as a clinically relevant biomarker to assist in antibiotic decision-making in patients with suspected bacterial infections. PCT levels may increase in response to bacterial infection and changes in disease activity; however, its diagnostic performance and clinical value are context-dependent, and interpretation of its results requires careful consideration of its limitations.

This structured narrative review aimed to examine the available evidence on PCT-guided antibiotic therapy, focusing on antibiotic decision-making and the clinical limitations affecting its use in practice. A literature search was conducted using PubMed, Scopus, and Web of Science. The review primarily included randomized controlled trials, meta-analyses, and widely recognized clinical guidelines.

Evidence supports the use of PCT mainly for guiding antibiotic discontinuation, helping to reduce antibiotic exposure without adversely affecting major clinical outcomes. In contrast, its role in guiding antibiotic initiation remains limited, particularly in sepsis, where early empirical antibiotic treatment is essential. Non-infectious inflammatory conditions can cause false-positive elevations in PCT. Conversely, early or localized infections may result in false-negative or lower PCT levels. The timing of measurement and variation in threshold values can also affect the interpretation of PCT results. Differences in the application of guideline recommendations across clinical settings may also influence its clinical utility.

PCT can support antimicrobial stewardship and antibiotic decision-making but should not be used as a standalone diagnostic tool. PCT results should always be interpreted within the clinical context, alongside clinical assessment and other available diagnostic information, while considering the limitations of the biomarker and the strength of the supporting evidence.

## Introduction and background

Bacterial infections are an important cause of morbidity and mortality worldwide, particularly among hospitalized and critically ill patients [1]. Differentiating bacterial from non-bacterial causes of illness remains a major diagnostic challenge despite advances in diagnostic tools and antimicrobial therapy. This diagnostic gap often leads to empirical antibiotic use, referring to treatment initiated before the causative pathogen is definitively identified, resulting in unnecessary exposure, adverse effects, and the global increase in antimicrobial resistance [2,3]. As a result, optimizing antibiotic use through antimicrobial stewardship, which aims to ensure appropriate antibiotic selection, dosing, duration, and discontinuation, has become an important focus of clinical practice.

To address these challenges, interest in host-response biomarkers has increased because they may improve diagnostic assessment and guide antibiotic decisions. Procalcitonin (PCT) is a precursor peptide of the hormone calcitonin. Its circulating concentration generally increases during bacterial infections and may be lower during many viral infections; however, PCT levels can also be influenced by non-infectious inflammatory conditions and other clinical factors [4,5]. Its kinetic profile and association with inflammatory activity have led to its incorporation into clinical algorithms for antibiotic therapy, particularly in respiratory tract infections and critical care [6].

Several randomized controlled trials and meta-analyses have evaluated PCT-guided antibiotic strategies. Overall, these studies showed that PCT can reduce unnecessary antibiotic exposure without negatively affecting major clinical outcomes [6,7]. However, the findings have not been entirely consistent across different clinical settings. The evidence supporting PCT-guided antibiotic discontinuation is stronger than the evidence supporting PCT-guided antibiotic initiation. Discontinuation refers to stopping antibiotics after treatment has been initiated, whereas initiation refers to the decision to start antibiotics. In addition, PCT results should always be interpreted with caution. Non-infectious inflammatory conditions, the timing of sample collection, infection burden and localization, host-related factors, and differences in assay thresholds can all influence PCT concentrations and affect clinical interpretation [8-10].

Despite the growing use of PCT, uncertainty remains regarding its interpretation and application across different clinical settings, particularly its role in antibiotic initiation versus discontinuation. Therefore, this review provides a critical overview of the available evidence on PCT-guided antibiotic therapy, focusing on its role in antimicrobial decision-making, clinical limitations, and practical challenges affecting its routine use in antimicrobial stewardship.

A structured narrative review of the available evidence on PCT-guided antibiotic therapy in adults with suspected or confirmed bacterial infections was conducted, focusing on the clinical utility and limitations of PCT in informing antibiotic decisions.

The literature search was conducted separately in PubMed/MEDLINE, Scopus, and Web of Science. The primary search covered publications from 2010 through 2025. The searches were conducted on May 21, 2026, in PubMed/MEDLINE; May 23, 2026, in Scopus; and May 27, 2026, in Web of Science. The search strategy focused on three main concepts: PCT; antibiotic decision-making, including antibiotic stewardship, initiation, discontinuation, and treatment duration; and the clinical contexts of sepsis and respiratory tract infections. Reference lists of relevant articles were also screened to identify additional publications, including important earlier publications providing biological or historical background.

Database-specific Boolean search strategies were used for PubMed/MEDLINE, Scopus, and Web of Science, with equivalent search concepts adapted to the syntax and field tags of each database.

Studies were eligible if they assessed the role of PCT as a tool to aid in antibiotic initiation, discontinuation, or other antibiotic decisions in adults. Randomized controlled trials, systematic reviews, meta-analyses, and clinical guidelines from different countries were given the greatest priority due to their high level of evidence. Observational studies were included when they offered valuable information on real-world practice or addressed questions not covered by interventional studies. 

Studies involving only experimental laboratory models, those that did not evaluate PCT as a biomarker, and studies conducted exclusively in pediatric populations were excluded. Publications written in languages other than English were not systematically reviewed, which may have introduced language bias. 

Following the removal of duplicate records, two reviewers independently screened the identified studies in two stages. Titles and abstracts were first assessed against the predefined inclusion and exclusion criteria, followed by the full-text assessment of potentially relevant records. Disagreements between the reviewers were resolved through discussion and consensus. The included studies were synthesized according to their clinical relevance, methodological quality, and consistency across different clinical settings.

As this was a narrative review, a formal systematic review framework, such as Preferred Reporting Items for Systematic Reviews and Meta-Analyses (PRISMA), and quantitative meta-analysis were not used. A formal risk-of-bias assessment was not performed because the review was designed as a narrative synthesis rather than a systematic review.

The included studies were grouped into three broad themes: the biological basis of PCT, clinical applications, and evidence-based limitations. Particular focus was placed on the role of PCT in antimicrobial initiation and discontinuation and on factors affecting its use in routine clinical practice.

## Review

Physiology and baseline expression

Under physiological conditions, the parafollicular C cells of the thyroid gland produce PCT, a 116-amino-acid precursor peptide of calcitonin [4]. In healthy individuals, blood PCT levels are generally very low or undetectable because the peptide is rapidly cleaved into calcitonin and inactive fragments [8]. This low baseline concentration provides a reference for interpreting pathological elevations.

Mechanism of elevation in bacterial infections

During bacterial infection, PCT production shifts from the thyroid to more diffuse extrathyroidal synthesis in tissues such as the liver, lungs, and adipose tissue [11,12]. This systemic upregulation is mainly driven by pro-inflammatory cytokines, including interleukin-6 (IL-6) and tumor necrosis factor-alpha (TNF-α), as well as direct stimulation by bacterial endotoxins [13]. PCT is relatively specific for bacterial infection. In viral infections, interferon-gamma (IFN-γ) generally suppresses PCT production, resulting in lower plasma levels [4]. This differential immune response provides a biological basis for using PCT to help distinguish bacterial from viral infections, although the distinction is not absolute and may be affected by host and disease factors.

Kinetics and clinical interpretation

PCT has favorable kinetic characteristics for clinical application. In response to bacterial infection, serum levels usually begin to increase within 4-6 hours, peak at approximately 12-24 hours, and then decline when the infection source is controlled and/or adequate antimicrobial treatment is provided [5]. Its half-life is approximately 24 hours, allowing serial measurements to reflect changes in inflammation over time [6]. These dynamic properties have led to algorithms that use both absolute values and temporal trends to guide antibiotic decisions. Trends in PCT levels are generally more informative than individual measurements. However, there is no strict linear relationship between infection severity and PCT level, and substantial inter-individual variation exists according to host factors, infection type, and disease progression [9,10]. Therefore, isolated PCT values, particularly in complex or high-risk situations, should not be used as the sole basis for clinical decision-making. 

Clinical applications of PCT

Respiratory Tract Infections

PCT-guided antibiotic therapy is best studied in the respiratory tract infection setting [6,14]. This is clinically relevant because bacterial and viral causes frequently overlap in lower respiratory tract syndromes, creating substantial uncertainty regarding antibiotic prescribing [6,15]. In this setting, PCT has been studied primarily as a means of limiting unnecessary antibiotic use rather than as a diagnostic test.

Evidence from randomized trials and pooled analyses indicates that PCT-based algorithms can reduce antibiotic initiation and shorten antibiotic treatment in acute respiratory tract infections, including community-acquired pneumonia, acute bronchitis, and exacerbations of chronic obstructive pulmonary disease [6,14,16]. In a patient-level meta-analysis, Schuetz et al. found that PCT-based care reduced antibiotic exposure and antibiotic-related adverse events without consistently increasing treatment failure or mortality [6]. These findings support the use of PCT as an antimicrobial stewardship tool in respiratory infections rather than as a standalone method for pathogen diagnosis.

However, this apparent benefit in respiratory infections should be considered in the context of case mix and baseline risk [6,14]. Most supportive evidence comes from outpatient, emergency department, and non-intensive care unit (non-ICU) populations with a lower pretest probability of severe bacterial disease, in whom withholding or shortening antibiotic therapy is generally safer [6,16]. This may partly explain why the impact of PCT is more apparent in respiratory infections than in more severe syndromes such as sepsis. Some of the observed benefit may therefore be related to the clinical setting in which the stewardship intervention is implemented rather than to the overall efficacy of the biomarker.

This is particularly important in community-acquired pneumonia. Although PCT may provide additional information, current major guidelines do not support using a low PCT level as a reason to delay antibiotics when clinical suspicion of bacterial pneumonia is high [17,18]. PCT is more useful for reassessment than for selecting initial treatment. Therefore, the evidence favors using PCT to support earlier discontinuation or shorter treatment duration rather than to exclude the need for initial therapy.

In general, the clinical usefulness of PCT in respiratory tract infections varies among patients. It appears most beneficial in patients with low to moderate illness severity, intermediate diagnostic uncertainty, and an opportunity to avoid unnecessary antibiotic exposure [6,14,18]. As disease severity increases, its role becomes less clear and should remain secondary to clinical evaluation, imaging, and risk stratification.

Sepsis and Critical Care

PCT-guided antibiotic therapy has been widely studied in critically ill patients, particularly those with suspected or established sepsis. Early treatment with broad-spectrum antibiotics remains a mainstay of management because delayed treatment is associated with higher mortality [19]. However, prolonged or inappropriate antibiotic use is also common in ICUs, contributing to antimicrobial resistance, drug-related toxicity, and secondary infections [20]. This dual challenge has generated interest in PCT as an antimicrobial stewardship tool. In contrast to respiratory tract infections, its primary role in critical care is not differential diagnosis at presentation but guidance of antibiotic discontinuation after treatment has been initiated. This distinction reflects the need for rapid treatment decisions in sepsis, followed by reassessment as additional clinical and biochemical data become available.

In the ICU setting, several randomized controlled trials have been conducted to assess the effectiveness of PCT-based protocols for antibiotic discontinuation, which are mostly based on absolute thresholds or relative reductions [7,21]. All these studies have shown that a PCT-based approach can shorten antibiotic treatment and cumulative exposure in critically ill patients [7,22]. Importantly, these benefits are most apparent when used in a structured protocol that includes serial measurements and not just a single measurement. 

One influential trial showed that PCT-guided discontinuation reduced antibiotic duration without compromising clinical outcomes [7]. Subsequent meta-analyses have supported shorter antibiotic courses with PCT-based approaches, although findings regarding mortality benefits have varied across studies [22,23]. Importantly, the magnitude of benefit appears closely related to adherence to the algorithm, which varies considerably across clinical settings. PCT has a limited role in guiding antibiotic initiation because treatment decisions in sepsis are time-sensitive and should not be delayed while awaiting biomarker results [19,24]. These findings support the greater role of PCT in reassessment and de-escalation than in initial antibiotic decision-making.

Moreover, critically ill patients frequently have non-infectious inflammatory conditions, such as major surgery, trauma, and organ dysfunction, which can increase PCT levels and reduce its specificity [10]. Renal dysfunction may also affect PCT kinetics and interpretation. In addition, heterogeneity in cutoff values, measurement timing, and protocol adherence limits the generalizability of trial results. Inconsistent clinician adoption and variation in institutional practice may further reduce the impact of PCT-guided strategies in real-world settings.

Overall, the available evidence indicates that PCT is a useful adjunct to antibiotic de-escalation and early discontinuation in clinically stable or improving patients, rather than a frontline tool for initiating antibiotic therapy. Optimal use requires integration with clinical judgment, serial evaluation, and structured stewardship protocols.

PCT in Antibiotic Initiation Versus Discontinuation

Across the clinical literature, a consistent finding is that PCT is more useful for stopping antibiotic therapy than for starting it [6]. This asymmetry reflects the different clinical circumstances in which these decisions are made. Patients with suspected severe bacterial infection, such as sepsis or severe pneumonia, may require immediate empirical antibiotic treatment. In these situations, treatment should not be delayed while awaiting a single PCT measurement [18,19].

Serial PCT measurements can provide additional information to support antibiotic de-escalation or discontinuation after treatment has been initiated and the patient has been clinically reassessed. At this stage, biomarker trends can be considered alongside changes in clinical status, which may provide more reliable guidance on treatment duration than on initial prescribing decisions [10,16].

The evidence for PCT-guided antibiotic initiation is strongest in selected low- to moderate-risk respiratory tract infections, particularly where the potential benefit of antibiotics is limited in patients with low PCT levels [6,25,26]. However, PCT should not be used as a standalone rule-out test, even in these settings. False-negative results may occur in early or localized infections and in atypical clinical presentations, limiting its value as an independent decision-making tool [10]. Current guidelines recommend early antibiotic treatment when clinical suspicion is high, regardless of the biomarker level, particularly in severe infection syndromes [18,19]. In contrast, evidence supporting PCT-guided antibiotic de-escalation is more consistent in both respiratory and critical care settings [10,16,22]. Common discontinuation strategies use absolute PCT thresholds or relative decreases over time. Frequently used thresholds include values below 0.25 ng/mL in less severe respiratory infections [10,13,16] and relative decreases of approximately 80% from peak levels [10,13,16]. These approaches have been associated with shorter antibiotic treatment and reduced overall antibiotic exposure without consistent evidence of adverse outcomes when combined with clinical evaluation [16,22,27].

More recent randomized evidence supports this pattern. The ADAPT-Sepsis trial showed a small but statistically significant reduction in antibiotic duration with a daily PCT-guided protocol, without breaching predefined noninferiority safety criteria [27]. The degree of benefit was modest and depended on protocol adherence and appropriate interpretation within the broader clinical context [17,27].

Overall, PCT-guided antibiotic therapy should be applied in a context-specific manner, with greater emphasis on reassessment and discontinuation than on treatment initiation. Its interpretation should remain integrated with clinical assessment and antimicrobial stewardship principles [17,19].

Clinical integration and considerations for practice

PCT is most useful as part of a broader decision-making approach rather than as a standalone diagnostic or therapeutic tool. PCT values should be interpreted alongside the patient's clinical presentation, imaging findings, microbiological data, and disease trajectory [19]. This integrated approach helps prevent excessive reliance on biomarker results and supports safe and effective antibiotic use.

PCT may be most useful when diagnostic uncertainty remains after the initial evaluation. In such situations, it can support risk stratification and reassessment over time. For example, if the patient's clinical condition stabilizes and inflammatory markers decline, a lower PCT level may support earlier discontinuation of antibiotics [6,7]. Conversely, a persistently elevated or increasing PCT level may indicate unresolved infection, inadequate source control, or complications.

Importantly, the success of PCT-guided strategies is closely related to the use of serial measurements. Dynamic trends may provide more clinically relevant information than individual measurements, particularly when considered alongside changes in vital signs, laboratory parameters, and organ function [8]. This temporal information allows PCT to support the monitoring of treatment response rather than serving as a static diagnostic test. However, implementation can be challenging because its clinical impact depends on the algorithm used and clinician adherence. Studies have reported substantial noncompliance with PCT-guided protocols for reasons that are not necessarily related to patient safety or disagreement with clinical judgment [28]. Therefore, the effectiveness of PCT in routine practice may differ from that observed in controlled trials.

Application of PCT should be selective and based on clinical context. In clinically stable or improving patients, serial PCT measurements may support the reassessment of the need for continued antibiotics. In patients with suspected severe bacterial infection or rapid deterioration, clinical assessment and timely treatment should take priority over PCT results [18,19].

In antimicrobial stewardship programs, PCT may support more appropriate antibiotic use by helping clinicians reassess treatment duration when clinically appropriate [6,7]. Its additional benefit may be limited in settings where antimicrobial stewardship practices are already well established and antibiotics are used appropriately. The clinical applications of PCT-guided antibiotic therapy across major clinical settings are summarized in Table 1. Further considerations regarding the diagnostic performance and interpretation of PCT are discussed in the following section.

**Table 1:** Clinical applications of PCT-guided antibiotic therapy across major clinical settings PCT: procalcitonin; RTI: respiratory tract infection

Clinical setting	Main clinical objective	Most appropriate use of PCT	Major limitations/cautions
Acute RTIs	Minimize the use of unnecessary antibiotics in patients with uncertain diagnosis	Aiding in the withholding of antibiotics in certain low-risk patients and, more regularly, reducing or stopping the antibiotics during reassessment	Not as reliable in severe pneumonia or high-risk presentations; in severe pneumonia or as high-risk presenters, use in conjunction with guideline-based care which still emphasizes clinical assessment
Community-acquired pneumonia	Reassess and steward treatment once it has been initiated	Primary use is for reassessment and for determining the duration of treatment (not for deciding whether to withhold initial antibiotics when clinical suspicion is high)	False-negative readings may happen early; initial treatment decisions are more dependent on disease severity and imaging findings
Sepsis and critical care	Optimize antibiotic duration after empiric therapy has started	Best for supporting de-escalation or discontinuation based on serial values or decline from peak level	Not suitable for delaying empiric therapy in suspected sepsis as the specificity of the test is lowered by non-infectious inflammation and organ dysfunction
Hospitalized adults with suspected sepsis in contemporary practice	Ensure safe, limited shortening of antibiotic courses in contemporary stewardship programs	Daily or serial therapy reassessment according to PCT levels	Effect size will vary depending on implementation fidelity and adherence to protocol as well as local prescribing patterns
Antibiotic initiation decisions	Decide whether antibiotics should be started at presentation	Limited role: can only assist in specific, low-risk respiratory presentations in combination with full clinical assessment	Low PCT values are not sufficiently reliable in severe infections, sepsis, or severe pneumococcal infections
Antibiotic discontinuation decisions	Determine whether ongoing antibiotic therapy can be stopped earlier	The majority of evidence-based use of PCT in settings	Needs repeated testing and adherence with algorithms and should be used in conjunction, not in place of, bedside assessment

Diagnostic performance and interpretive considerations

Despite the large number of studies evaluating PCT as a biomarker for bacterial infection, its diagnostic performance is highly context-dependent. Meta-analyses suggest that PCT has moderate sensitivity and specificity for systemic bacterial infections, with better performance in sepsis and bacteremia than in localized bacterial infections [29,30]. Therefore, its value lies more in supporting clinical probability assessment than in serving as a definitive confirmatory diagnostic test.

In respiratory tract infections, PCT has demonstrated utility in distinguishing bacterial from viral causes, particularly among patients with low to moderate disease severity [31]. However, its discriminatory power is reduced in more severe disease, when systemic inflammation may no longer be driven solely by infection. Similarly, among critically ill patients, high PCT levels may indicate bacterial infection but are too nonspecific to distinguish systemic inflammatory response syndrome (SIRS) and sepsis from other inflammatory responses [30].

Interpretation of PCT is influenced by the selected threshold and the timing of measurement. Interventional trials have used common cutoffs of 0.25 ng/mL in ambulatory or lower-risk settings and 0.5 ng/mL in critically ill patients, but these should be considered operational tools rather than absolute diagnostic thresholds [21]. Single measurements generally have limited diagnostic reliability. Although absolute values provide information about disease status, serial measurements can track changes over time and help assess inflammatory activity or treatment response [8]. PCT should therefore be considered part of a comprehensive clinical evaluation rather than a standalone diagnostic procedure.

Meta-analyses have demonstrated the diagnostic utility of PCT for bacterial infection. Self et al. reported a sensitivity of approximately 77% and a specificity of 79% for diagnosing sepsis [29]. Kamat et al. reported similar findings for bacteremia, with a sensitivity of approximately 76% and a specificity of 69% [30]. These findings support the use of PCT as a supplementary tool alongside primary diagnostic assessment rather than as an independent diagnostic method.

Commonly used PCT cutoffs come from interventional trials and stewardship algorithms. They should be viewed as intervention-specific thresholds rather than generalizable diagnostic cutoffs validated by clinical outcome. Furthermore, serial trends carry more information than isolated measurements, especially when used as justification for stopping antibiotics rather than withholding treatment [6,7,13]. Common PCT thresholds and their clinical interpretation are summarized in Table 2.

**Table 2:** Common PCT thresholds and context-based clinical interpretation PCT: procalcitonin; ICU: intensive care unit

PCT value or trend	Clinical interpretation	Common clinical context	Potential antibiotic implication	Key caution
<0.1 ng/mL	Very low likelihood of significant systemic bacterial infection	Low-risk respiratory tract infection, ambulatory or primary care settings	Strongly discourages antibiotic use in carefully selected patients	Should not be used alone to exclude early infection, localized infection, or severe bacterial disease
0.1 to <0.25 ng/mL	Bacterial infection less likely	Lower respiratory tract infections with low to moderate severity	Antibiotics may be discouraged or withheld when clinical suspicion is low and follow-up is reliable	Not recommended as the sole rule-out threshold for severe pneumonia, sepsis, or immunocompromised patients
0.25 to <0.5 ng/mL	Intermediate probability; bacterial infection possible	Emergency department or inpatient respiratory infections	Requires integration with symptoms, imaging, microbiology, and illness severity before deciding on treatment	This range is indeterminate and should not be used to make decisions alone
≥0.5 ng/mL	Higher likelihood of systemic bacterial infection	Hospitalized patients, ICU settings, suspected sepsis	Supports antibiotic therapy when consistent with clinical findings	High levels can also be seen with surgery, trauma, shock, or other non-infectious inflammatory conditions
Very high or rising values over time	Ongoing inflammatory response, possible uncontrolled infection, inadequate source control, or treatment failure	Sepsis, ICU, severe bacterial infection	May support further diagnostic reassessment rather than automatic antibiotic escalation	Persistent elevation is not specific and should be interpreted in conjunction with overall clinical presentations
Decline of approximately ≥80% from the peak value	Favorable biomarker response to treatment	ICU and hospitalized patients receiving serial PCT monitoring	Commonly used criterion to support antibiotic discontinuation or de-escalation	Trend-based interpretation is more useful than isolated values
Absolute value <0.25 ng/mL during follow-up	Low residual likelihood of ongoing clinically significant bacterial infection in less severe respiratory illness	Respiratory tract infections, non-ICU settings	May support early discontinuation of antibiotics	More applicable in lower-risk patients than in severe or complicated infection
Absolute value <0.5 ng/mL during follow-up	Reduced likelihood of ongoing systemic bacterial infection in critical illness	ICU, sepsis reassessment	Often used as a discontinuation threshold in critically ill patients	Should not exclude ongoing hemodynamic instability, an uncontrolled source of infection, or new signs of infection

Evidence-based limitations of PCT

False-Positive Elevations in Non-infectious Conditions

One of the major limitations of PCT is its lack of absolute specificity for bacterial infection. High PCT levels can also be seen in various non-infectious inflammatory conditions such as major surgery, trauma, burns, cardiogenic shock, and severe systemic stress in which cytokine-mediated pathways activate PCT production in the absence of microorganisms [8,10]. Recent evidence further supports this limitation. A modern study reported that in clinical practice, high levels of PCT are often found in non-infectious inflammatory states, including critically ill and postoperative patients, decreasing the specificity of the test. Furthermore, analyses performed in the ICU also support the fact that non-infectious systemic inflammation is a significant cause of false-positive elevation of PCT [6,10]. This limitation is particularly relevant in critically ill patients, in whom multiple overlapping inflammatory processes are common. 

False-Negative Results in Early, Localized, or Atypical Infection

Low PCT values do not rule out bacterial infection. In early infection, PCT elevation may be undetectable during an initial lag period, potentially leading to falsely reassuring results if testing is performed too early. Localized infections, such as abscesses or compartmentalized infections, may also produce a limited systemic response and therefore may not raise PCT sufficiently. In addition, host factors and atypical pathogens can influence PCT responses [32]. A systematic review found that PCT has moderate sensitivity for distinguishing bacterial from non-bacterial infections, particularly in the early stages of disease [10]. These findings further support the limitation of low PCT levels in excluding early or localized bacterial infection.

Variability in Cutoff Thresholds and Lack of Standardization

PCT cutoffs vary, making interpretation challenging. Common thresholds, including 0.25 ng/mL and 0.5 ng/mL, are derived from clinical trials and are not definitive diagnostic thresholds [6]. Variability also arises from differences in assay methodology, patient populations, and clinical settings. Differences in PCT thresholds across studies represent an important limitation. A systematic review found that cutoff values varied across clinical trials and real-world practice, suggesting that PCT thresholds are influenced substantially by study protocols and clinical context [32].

Dependence on Timing and Serial Measurement

The timing of PCT measurement is clinically important, and serial changes in PCT are often more informative than single values. A single measurement does not provide a dynamic view of the biomarker response and therefore has limited value for clinical decision-making. Serial measurements can provide better information about disease progression and response to therapy [8]. Recent evidence similarly indicates that monitoring PCT over time provides more reliable guidance for antibiotic therapy than relying on a single measurement [10,11].

Heterogeneity of Clinical Evidence and Study Design

Evidence for PCT-guided therapy is mixed. Wide variation in study populations, disease severity, clinical context, testing frequency, and adherence to PCT-guided protocols makes direct comparison difficult. These differences also limit the applicability of findings to other healthcare settings. Furthermore, many studies were conducted under controlled research conditions with standardized protocols that may not fully capture the variability of routine clinical practice. Therefore, reported benefits should be interpreted with caution [6,22].

Implementation Barriers in Real-World Practice

The success of PCT-based approaches depends heavily on adherence to recommended guidelines and protocols. In clinical practice, adherence is not always optimal because clinicians may deviate from PCT-based recommendations because of safety concerns, severe illness, or conflicting clinical findings [25,28]. Variations in institutional policies, availability of rapid PCT testing, and cost may also limit adoption. Recent evidence suggests that although these strategies are safe, their impact on antibiotic consumption may be smaller when antimicrobial stewardship programs are already well established. For example, the ADAPT-Sepsis trial demonstrated a modest but statistically significant reduction in antibiotic use with PCT-guided treatment [27].

The Impact of Patient-Specific Factors

Patient-specific factors can influence PCT levels and complicate interpretation. For example, PCT may remain elevated in patients with renal dysfunction because of reduced clearance, even in the absence of infection. Conversely, immunocompromised patients may have a limited inflammatory response and low PCT levels despite active infection [9,10]. These variations may affect diagnostic reliability and underscore the importance of interpreting PCT in the context of the patient's overall clinical picture.

Risk of Over-Reliance and Misinterpretation

Over-reliance on PCT results without considering the patient's clinical presentation can lead to inappropriate treatment decisions. Patients with elevated PCT levels may receive antibiotics unnecessarily or for longer than needed, whereas patients with serious infections may experience delayed treatment despite low PCT levels [13]. Evidence supports using PCT alongside clinical assessment and other laboratory findings [10,11]. Therefore, PCT should remain an adjunct to clinical judgment.

Overall evidence and clinical context

This review evaluated the current evidence supporting the role of PCT in antibiotic decision-making and examined the main limitations affecting its clinical use. The evidence indicates that PCT can reduce unnecessary antibiotic use without compromising patient safety, although its utility varies according to clinical context. Important limitations include imperfect diagnostic specificity, variation in cutoff thresholds, and differences in clinical implementation. A key finding is that PCT is more effective for supporting antibiotic discontinuation than initiation, particularly because discontinuation decisions are generally made after clinical reassessment and evaluation of biomarker trends.

PCT performance varies across clinical settings. In respiratory tract infections, PCT-guided strategies can reduce antibiotic use in selected patients, particularly those with lower disease severity. In critically ill patients, PCT-guided protocols may shorten antibiotic treatment after empirical therapy has been initiated, but current evidence does not support withholding antibiotics based on PCT alone. These differences reflect variation in disease severity, clinical risk, timing of decisions, and diagnostic uncertainty.

Important limitations include variability in cutoff values, biological factors that may cause false-positive or false-negative results, and differences between controlled trials and routine clinical practice. The effectiveness of PCT-guided strategies may also depend on protocol adherence and the availability of appropriate antimicrobial stewardship practices. Therefore, PCT should be interpreted alongside clinical findings and other diagnostic information rather than used as an independent diagnostic or treatment decision tool.

Limitations

This review has several limitations. First, the narrative design may introduce subjectivity in the selection and interpretation of the available evidence. Although efforts were made to include relevant and high-quality studies, a formal risk-of-bias assessment was not conducted. Second, the heterogeneity among studies regarding patient populations, clinical settings, and PCT-guided protocols may limit the generalizability of the findings. Finally, the inclusion of only English-language publications may have introduced potential language bias. 

Future directions

Future research should focus on improving PCT-guided strategies through integration with clinical prediction models and emerging technologies, including artificial intelligence. Combining PCT with other biomarkers, such as C-reactive protein and novel host-response markers, may provide a more comprehensive assessment of infection risk and improve antibiotic decision-making. Further studies are also needed to establish clinically relevant cutoff values for different patient populations and to evaluate effective strategies for implementing PCT-guided approaches in routine clinical practice.

## Conclusions

Current evidence indicates that PCT can support antibiotic decision-making when interpreted alongside clinical judgment rather than used as a standalone marker. Its role is better established in guiding antibiotic discontinuation, where PCT-guided strategies can reduce antibiotic exposure and treatment duration without compromising major clinical outcomes. In contrast, its role in antibiotic initiation is more limited, particularly in high-risk situations where early empirical treatment is required. Important limitations include false-positive elevations in non-infectious inflammatory conditions, low sensitivity in early or localized infections, variability in diagnostic thresholds, and challenges in routine implementation. Therefore, PCT is most valuable when incorporated into structured antimicrobial stewardship strategies and interpreted in the context of the overall clinical picture. Future research should focus on optimizing PCT-guided strategies, improving their implementation in routine practice, and integrating PCT with other diagnostic approaches.
